# Supplementary material for: A molecular classification of human mesenchymal stromal cells
Source: PeerJ. 2016 Mar 24;4:e1845. doi: 10.7717/peerj.1845 (PMC4811172; doi:10.7717/peerj.1845)
Supplement: Supplemental Information 1 [file peerj-04-1845-s001.pdf]

An online resource for the Molecular Classification of Human Mesenchymal Stromal Cells

Florian Rohart[1], Elizabeth Mason[1], Nicholas Matigian[1], Rowland Mosbergen[1], Othmar Korn[1], Tyrone Chen[1], Suzanne Butcher[1], Jatin Patel[2], Kerry Atkinson[2], Kiarash Khosrotehrani[2,3], Nicholas M Fisk[2,4], Kim-Anh Lê Cao[3] and Christine A Wells[1,5*]

[1] Australian Institute for Bioengineering and Nanotechnology, The University of Queensland, Brisbane, QLD Australia 4072

[2] The University of Queensland Centre for Clinical Research, Herston, Brisbane, Queensland, Australia, 4029

[3] The University of Queensland Diamantina Institute, Translational Research Institute, Woolloongabba, Brisbane QLD Australia, 4102

[4] Centre for Advanced Prenatal Care, Royal Brisbane & Women's Hospital, Herston, Brisbane, Queensland, Australia, 4029

[5] Institute for Infection, Immunity and Inflammation, College of Medical, Veterinary & Life Sciences, The University of Glasgow, Scotland, UK G12 8TA

*Correspondence to: Christine Wells, c.wells@uq.edu.au

Contents of Supplemental Files:
Extended methods:

- Design of test and training datasets
- Pre-processing of the data
- Statistical analysis
    - Notations
    - A sparse multivariate procedure for discriminant analysis and gene selection
    - Constrained sPLS-DA
    - Prediction with sPLS-DA
- The signature tool
    - Random subsampling
    - Cross-Validation
    - Choosing the number of sPLS-DA components
    - Choosing the number of stable genes per component
    - Definitive assignation of a test sample to the MSC or non-MSC class
- Benchmarking the signature
    - The MSC signature outperforms common MSC surface markers
    - Batch effect and platform effect
- Methodological References

Supplemental Figures and Tables:

Supplemental Figure S1: Box-whisker plots of the average expression of 16 non-differentially expressed common cell surface MSC markers across samples in the training dataset. MSC (n=115), non-MSC (n=510). Accompanies Figure 1 and Table 1.

Supplemental Figure S2: Overview of experimental or platform batch on the MSC classification. The first two components are plotted on a PLS-DA scatter plot and training samples colored by (A) the Stemformatics dataset ID (see annotation legend below); (B) The microarray platform (C) the platform manufacturer. (D) non-MSC sample annotation.(E) MSC sample annotation. Circles are MSC, triangles are non-MSC. Accompanies Manuscript Figure 2.

Supplemental Figure S3: Accompanies Manuscript Figure 3. **S3A**: Box-whisker plot of Component 2, 3 and 4 signature genes. **S3B**: Differential expression analysis of bone marrow MSC vs other MSC classes. Heatmap of genes in Supplementary Table S6, rows are genes, columns are MSC samples. Legend (top) indicates tissue source, (bottom) indicates fetal vs adult tissue. **S3C** GO term enrichment for DEG genes.

Table S1. Markers used in the common MSC immunophenotyping panel. Accompanies Figure 1 and Table 1.

Table S2. (auxiliary spreadsheet). Datasets in the training set; accompanies Figure 2 and methods.

Table S3. Overview of the samples included in the training set, accompanies Figure 2 and methods.

Table S4. Members of the MSC signature network, seeded from component 1 genes and first-order interaction partners (BioGRID). Column 1 provides information on Gene Symbol (and ENSEMBL ID for seed members). Column 2 indicates Component or network membership and Probability of selection derived from sPLS-DA analysis. Disease annotations taken from the Mendelian Inheritance in Man (MIM) database. Accompanies Figure 2.

Table S5. (auxiliary spreadsheet). Datasets that contributed to the testing set and result of the Rohart test. Accompanies Figure 3. MSC status is unknown in the absence of phenotype data provided in the manuscript.

Table S6. (auxiliary spreadsheet). Top Differentially expressed genes between MSC from bone marrow compared to other sites. Linear mixed model, FDR adjusted P<0.01. Top Differentially expressed genes between MSC from different sites. Linear mixed model, FDR adjusted P<0.01.

Dataset References

Figure S1: Box-whisker plots of the average expression of 16 non-differentially expressed common cell surface MSC markers across samples in the training dataset, depending on cell type. Box-whisker plots showing median expression, and 25th and 75th percentiles. MSC (n=115), non-MSC (n=510). Accompanies Figure 1 and Table 1.

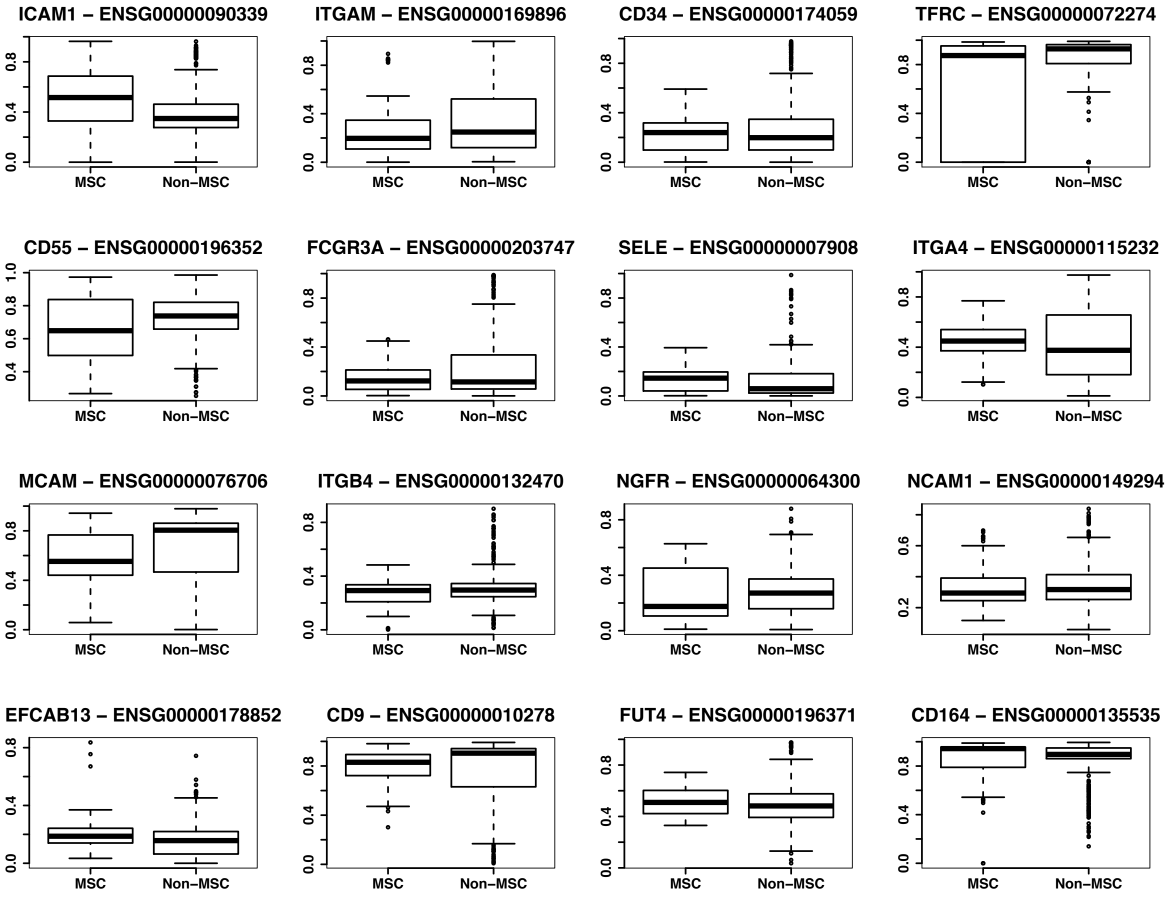

Implementation in Stemformatics

All primary data are available from the Stemformatics stem cell resource. The underlying code for the statistical test is available as BootsPLS in the CRAN repository, and we have also made available the d3 code for the interactive MSC graph implemented in Stemformatics via the BioJS framework at http://biojs.io/d/biojs-vis-rohart-msc-test

Figure S2: Overview of experimental or platform batch on the MSC classification. The first two components are plotted on a PLS-DA scatter plot and training samples colored by (A) the Stemformatics dataset ID (see annotation legend below); (B) The microarray platform (C) the platform manufacturer. (D) non-MSC sample annotation.(E) MSC sample annotation. Circles are MSC, triangles are non-MSC. Accompanies Manuscript Figure 2.

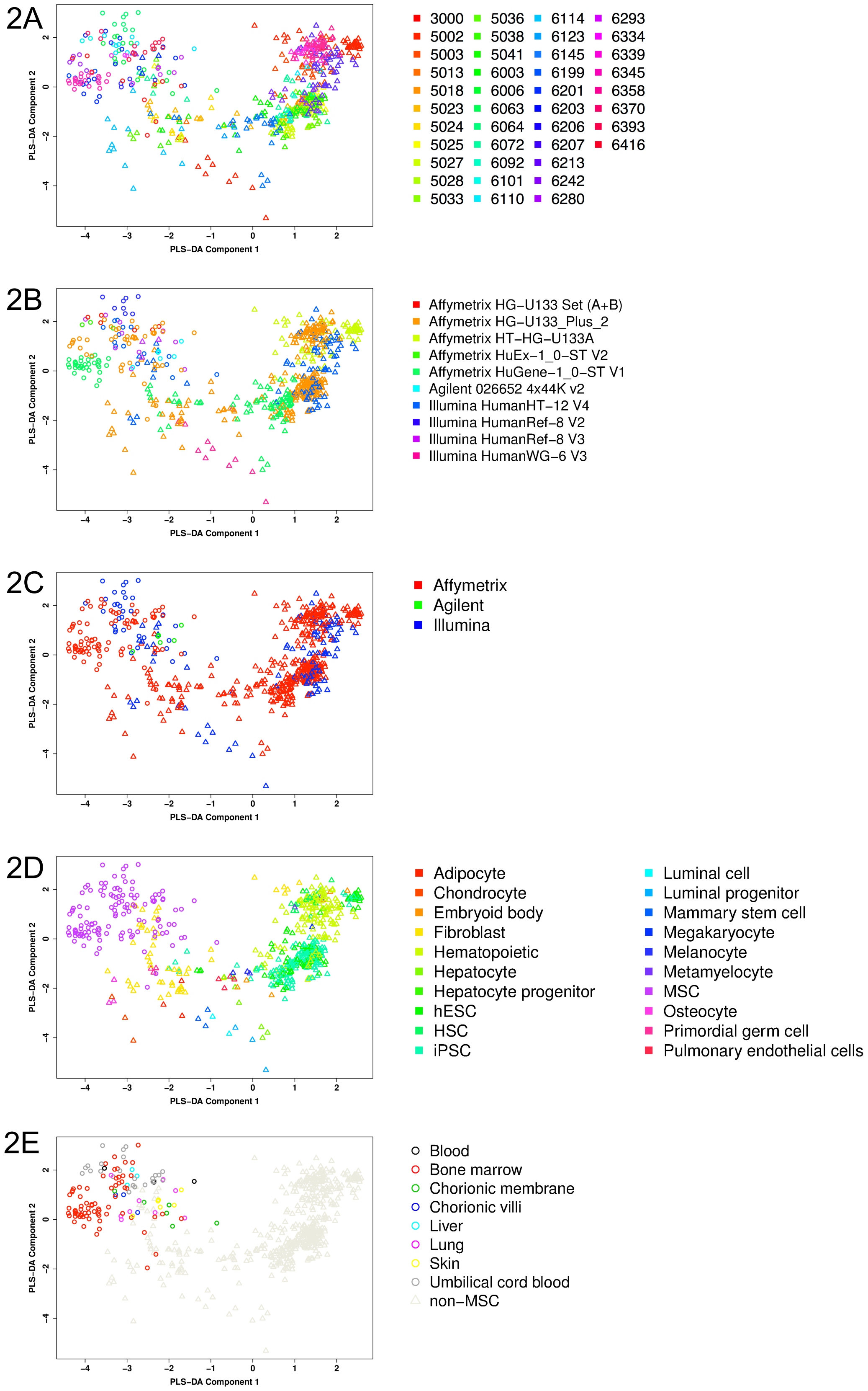

Figure S3: Accompanies Manuscript Figure 3. **S3A**: Box-whisker plot of Component 2, 3 and 4 signature genes. **S3B**: Differential expression analysis of bone marrow MSC vs other MSC classes. Heatmap of genes in Supplementary Table S6, rows are genes, columns are MSC samples. Legend (top) indicates tissue source, (bottom) indicates fetal vs adult tissue. **S3C** GO term enrichment for DEG genes.

3A

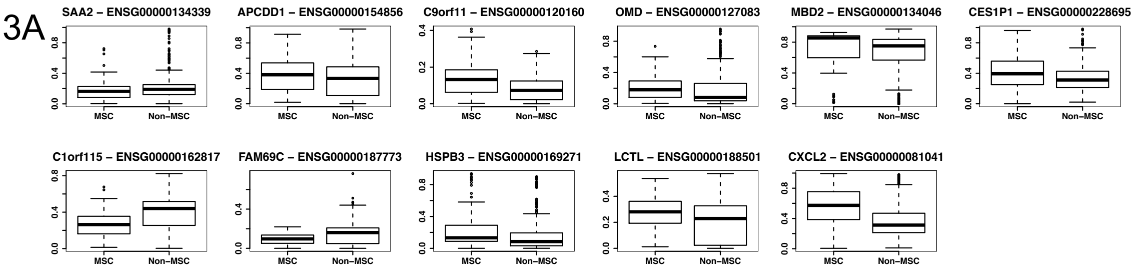

3B

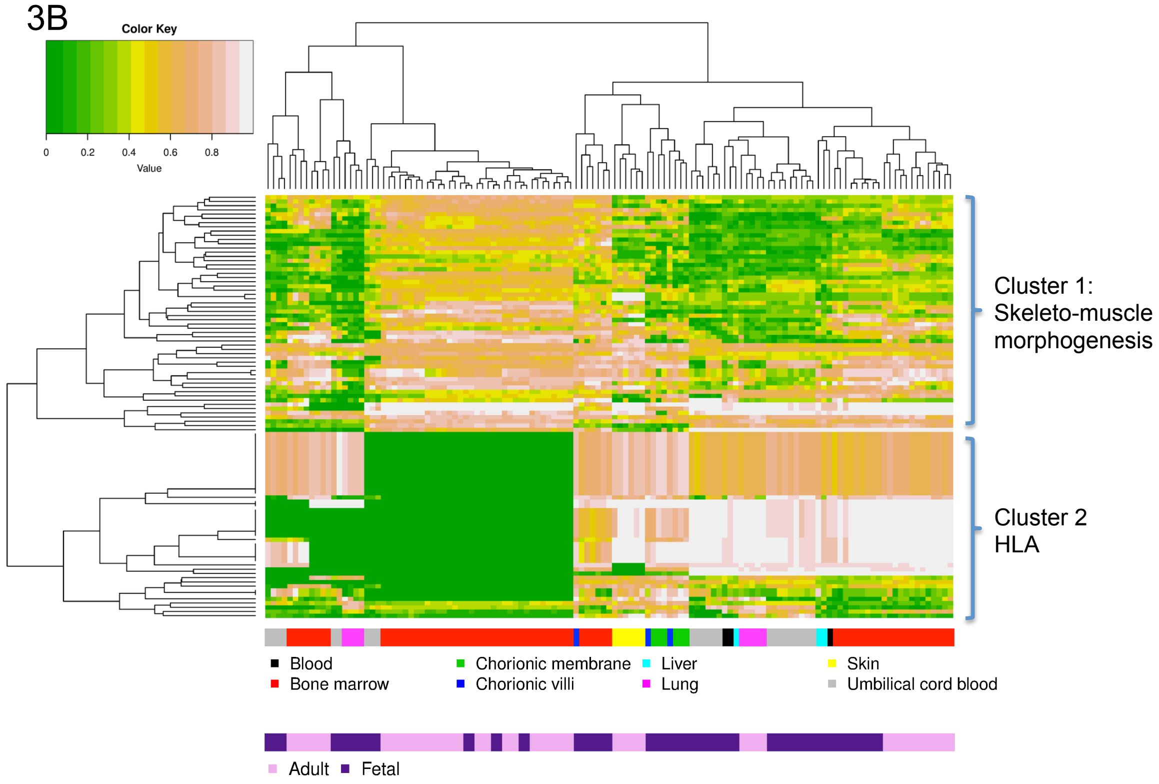

3C

Cluster	Annotation	Corrected P<value	Key genes
1 (56%)<br>Highest BM-MSC	Skeleto-Muscle Morphogenesis<br><br>DNA BINDING	GO:0001501 *P=1.2E-4*<br>GO:0007275<br>*P=4.8E-3*<br>GO:0043565<br>*P=4.1E-3*	COMP, DLX5, EBF3, EYA1, EYA2, FOXC1, GLIS1, HOXA9, HOXA10, HOXC9, HOXD1, IGF2, KAZALD1, MID1, MEOX2, NKX3.2, PRDM16, ZNF423
2 (44%)<br>Lowest BM-MSC	MHC-1<br>DNA BINDING	GO:0019885<br>*P=1.5E-16*<br>GO:0043565<br>*P=8.4E-3*	TAP1, TAP1AS, HLA-B, HLA-C, HLA-H , FOXF1, HOXC4, HOXC6.

Table S1. Markers used in the common MSC immunophenotyping panel

Gene Name	Aliases	Ensembl ID	Description
ALCAM	CD166 MEMD	ENSG00000170017	activated leukocyte cell adhesion molecule
ANPEP	APN CD13 GP150 LAP1 P150 PEPN	ENSG00000166825	alanyl (membrane) aminopeptidase
CD164	MGC-24 MUC-24 endolyn	ENSG00000135535	CD164 molecule, sialomucin
CD34		ENSG00000174059	CD34 molecule
CD44	CDW44 CSPG8 ECMR-III HCELL HUTCH-I IN LHR MC56 MDU2 MDU3 MIC4 Pgp1	ENSG00000026508	CD44 molecule (Indian blood group)
CD55	CR CROM DAF TC	ENSG00000196352	CD55 molecule, decay accelerating factor for complement (Cromer blood group)
CD68	GP110 LAMP4 SCARD1	ENSG00000129226	CD68 molecule
CD9	BTCC-1 DRAP-27 MIC3 MRP-1 TSPAN-29 TSPAN29	ENSG00000010278	CD9 molecule
CR1	C3BR C4BR CD35 KN	ENSG00000203710	complement component (3b/4b) receptor 1 (Knops blood group)
EFCAB13	C17orf57	ENSG00000178852	EF-hand calcium binding domain 13
ENG	CD105 END HHT1 ORW ORW1	ENSG00000106991	endoglin
FCGR3A	CD16 CD16A FCG3 FCGR3 FCGRIII FCR-10 FCRIII FCRIIIA IGFR3	ENSG00000203747	Fc fragment of IgG, low affinity IIIa, receptor (CD16a)
FUT4	CD15 ELFT FCT3A FUC-TIV FUTIV LeX SSEA-1	ENSG00000196371	fucosyltransferase 4 (alpha (1,3) fucosyltransferase, myeloid-specific)
ICAM1	BB2 CD54 P3.58	ENSG00000090339	intercellular adhesion molecule 1
ITGA4	CD49D IA4	ENSG00000115232	integrin, alpha 4 (antigen CD49D, alpha 4 subunit of VLA-4 receptor)
ITGA5	CD49e FNRA VLA5A	ENSG00000161638	integrin, alpha 5 (fibronectin receptor, alpha polypeptide)
ITGAM	CD11B CR3A MAC-1 MAC1A MO1A SLEB6	ENSG00000169896	integrin, alpha M (complement component 3 receptor 3 subunit)
ITGB1	CD29 FNRB GPIIA MDF2 MSK12 VLA-BETA VLAB	ENSG00000150093	integrin, beta 1 (fibronectin receptor, beta polypeptide, antigen CD29 includes MDF2, MSK12)
ITGB4	CD104	ENSG00000132470	integrin, beta 4
KIT	C-Kit CD117 PBT SCFR	ENSG00000157404	v-kit Hardy-Zuckerman 4 feline sarcoma viral oncogene homolog
MCAM	CD146 MUC18	ENSG00000076706	melanoma cell adhesion molecule
MME	CALLA CD10 NEP SFE	ENSG00000196549	membrane metallo-endopeptidase
NCAM1	CD56 MSK39 NCAM	ENSG00000149294	neural cell adhesion molecule 1
NGFR	CD271 Gp80-LNGFR TNFRSF16 p75(NTR) p75NTR	ENSG00000064300	nerve growth factor receptor
NT5E	CD73 E5NT NT NT5 NTE eN eNT	ENSG00000135318	5'-nucleotidase, ecto (CD73)
PDGFRB	CD140B JTK12 PDGFR PDGFR-1 PDGFR1	ENSG00000113721	platelet-derived growth factor receptor, beta polypeptide
PECAM1	CD31 CD31/EndoCAM GPIIA' PECA1 PECAM-1 endoCAM	ENSG00000261371	platelet/endothelial cell adhesion molecule 1
PTPRC	B220 CD45 CD45R GP180 L-CA LCA LY5 T200	ENSG00000081237	protein tyrosine phosphatase, receptor type, C
SELE	CD62E ELAM ELAM1 ESEL LECAM2	ENSG00000007908	selectin E
TFRC	CD71 T9 TFR TFR1 TR TRFR p90	ENSG00000072274	transferrin receptor (p90, CD71)
THY1	CD90	ENSG00000154096	Thy-1 cell surface antigen
VCAM1	CD106 INCAM-100	ENSG00000162692	vascular cell adhesion molecule 1

Table S3: Overview of the samples included in the training set

Tissue group	Number of samples	MSC or non-MSC designation
iPSC reprogramming series	150	non-MSC
MSC	125	MSC
hESC	103	non-MSC
Fibroblast	54	non-MSC
T-cell	36	non-MSC
B-cell	18	non-MSC
HSC	17	non-MSC
Monocyte	17	non-MSC
Hepatocyte	13	non-MSC
Embryoid body	8	non-MSC
Myeloid progenitor	7	non-MSC
GMP	6	non-MSC
Erythroid cell	5	non-MSC
Pulmonary endothelial cells	4	non-MSC
Adipocyte	3	non-MSC
Band cell	3	non-MSC
Blood progenitor	3	non-MSC
Chondrocyte	3	non-MSC
Common Myeloid Progenitor	3	non-MSC
Early PM	3	non-MSC
Late PM	3	non-MSC
Luminal cell	3	non-MSC
Luminal progenitor	3	non-MSC
Lymphocyte	3	non-MSC
Macrophages	3	non-MSC
Mammary stem cell	3	non-MSC
Melanocyte	3	non-MSC
Metamyelocyte	3	non-MSC
Natural killer cell	3	non-MSC
Neural progenitor	3	non-MSC
Osteocyte	3	non-MSC
PMN	3	non-MSC
Primordial germ cell	3	non-MSC
Granulocyte	2	non-MSC
Hepatocyte progenitor	2	non-MSC
Megakaryocyte/Erythroid Progenitor	2	non-MSC
MPP	2	non-MSC
Myelocyte	2	non-MSC
Basophil	1	non-MSC
BC	1	non-MSC
Dendritic cell	1	non-MSC
Eosinophil	1	non-MSC
Megakaryocyte	1	non-MSC

Table S4: Members of the MSC signature network, seeded from component 1 genes and first-order interaction partners (BioGRID). Column 1 provides information on Gene Symbol (and ENSEMBL ID for seed members). Column 2 indicates Component or network membership and Probability of selection derived from sPLS-DA analysis. Disease annotations taken from the Mendelian Inheritance in Man (MIM) database. Accompanies Figure 2.

Gene symbol (ENSMBL Gene ID)	Component (Probability of selection)	Network criteria	Function	Associated Mendelian disease
TM4SF1 ENSG00000169908	1 (P=0.42) Network member	Seed	A transmembrane protein expressed on endothelial cells that facilitates cell migration and vessel formation (Zukauskas et al., 2011).	
BGN ENSG00000182492	1 (P=0.47) Network member	Seed	Extracellular proteoglycan that binds BMP4, TGFb and regulates osteoblast differentiation. Mice lacking biglycan are osteoporotic (Xu et al., 1998).	Ehlers-Danlos Syndrome (Corsi et al., 2002)
PRR16 ENSG00000184838	1 (P=0.57) Network member	Seed	Regulates cell size by regulating translation of mitochondrial proteins (Yamamoto et al., 2014).	
VCAM1 ENSG00000162692	1 (P=0.60) Network member	Seed	Plasma membrane, heavily glycosylated adhesion molecule. Interacts with integrins ITGA4/ITGB1 to facilitate adhesion of leukocytes to endothelial cells. Known MSC marker, thought to be important for immunomodulation of T-cells (Ren et al., 2010). Has a role the neural stem cell niche (Kokovay et al., 2012).	
SCRG1 ENSG00000164106	1 (P=0.65) component only	Seed	May have a role in bone and cartilage formation(Ochi, Derfoul & Tuan, 2006).	
KCNE4 ENSG00000152049	1 (P=0.66) component only	Seed	Potassium channel	Atrial fibrillation and cardiac arrhythmia (Crump & Abbott, 2014)
SPINT2 ENSG00000167642	1 (P=0.66) Network member	Seed	Secreted protease inhibitor, inhibits the enzyme that activates hepatocyte growth factor. Inhibits kallikrein. Mutations in SPINT2 lead to congenital tufting enteropathy, which is a disorder of the small intestine with villous atrophy and crypt hyperplasia.	Congenital tufting enteropathy (Salomon et al., 2014)
CRYAB / HSPB5 ENSG00000109846	1 (P=0.67) Network member	Seed	Immunomodulatory chaperone protein. Forms stable complexes that prevent protein aggregation.	Cardiomyopathy (MIM615184), Cataract (MIM 613763), Myopathy (MIM 608810; MIM 613869)
HSPB6 ENSG00000004776	1 (P=0.70) Network member	Seed	Chaperone protein involved in muscle contractile function. Cardioprotective. Reviewed in (Dreiza et al., 2010)	
PDGFRB ENSG00000113721	1 (P=0.76) Network member	Seed	Plasma membrane receptor tyrosine kinase. Required for pericyte recruitment during vascular development (Lindblom et al., 2003) Required for blood brain barrier (Armulik et al., 2010). Binds heparin sulfate proteoglycans to limit PDGF diffusion (Abramsson et al., 2007). Marks Mesenchymal stem cell fraction in bone marrow (Koide et al., 2007)(Koide et al., 2007)	Basal ganglia calcification (MIM615007), Myeloproliferative disorder with eosinophilia (MIM131440), Myofibromatosis (MIM228550)
APCDD1L ENSG00000198768	1 (P=0.80) Network member	Seed	Transmembrane protein of unknown function.	
NUPR1 ENSG00000176046	1 (P=0.82) Component only	Seed	Negative regulator of histone acetyltransferase (HAT) activity during DNA repair (Gironella et al., 2009)	Required for pancreatic cancer TGF-beta- and EGF -induced cell migration, invasion, anchorage-independent growth, and ability to form tumors in nude mice.(Sandi et al., 2011)
SRPX2 ENSG00000102359	1 (P=0.82) Component only	Seed	Involved in synaptic formation	Has a role in Polymicrogyria, speech disorders and epilepsy (Royer-Zemmour et al., 2008; Sia,

				Clem & Huganir, 2013)
CCDC80 / Equarin / Dro1 ENSG00000091986	1 (P=0.83) Network member	Seed	Extracellular protein that binds heparan sulfate proteoglycans. Binds Syndecan3 to regulate cell-matrix attachment(Song et al., 2013). CCDC80 regulates lens formation in the developing eye (Jarrin, Pandit & Gunhaga, 2012) Binds growth factors including FGF in the extracellular matrix(Jarrin, Pandit & Gunhaga, 2012; Song et al., 2012). Is an important component of the bone marrow stroma and loss prevents HSC niche development (Charbord et al., 2015). Mouse homolog Dro-1 is a tumour suppressor: CCDC80 (Dro-1) KO mice develop thyroid adenomas and ovarian carcinomas(Leone et al., 2015).	KO mice exhibit mild late-onset obesity, hyperphagia Regulates adipogenesis (Tremblay et al., 2009, 2012) Susceptibility loci for atopic dermatitis in a Japanese GWAS.(Hirota et al., 2012)
DDR2 ENSG00000162733	1 (P=0.85) Network member	Seed	Transmembrane receptor tyrosine kinase that interacts with fibrillar collagen and is required for the proliferation of mesodermal cells. Mice lacking Ddr2 have dwarfism and are infertile(Kawai et al., 2012). DDR2 regulates the size of long bones and the amount of body fat(Kawai et al., 2014)<br>Reviewed in (Leitinger, 2014)	Spondylometaepiphyseal dysplasia, short limb-hand type (MIM 271665)
ADAMTSL1 ENSG00000178031	1 (P=0.91) Component only	Seed		
VEGF-c ENSG00000150630	1 (P=0.92) Component only	Seed		Lymphedema, hereditary, ID, MIM615907
BDKRB1 ENSG00000100739	1 (P=0.93) Component only	Seed	The B1 receptor is synthesized de novo following tissue injury and mediates hyperalgesia in animal models of chronic inflammation.	
EFEMP2 / Fibulin4 ENSG00000172638	1 (P=0.97) Network member	Seed	Secreted protein, forms elastic fibers in connective tissues, binds and regulates availability of TGFb(Renard et al., 2010).	Cutis laxia type I (MIM604633) Lethal osteogenesis imperfecta-like condition with cutis laxa and arterial tortuosity(Erickson, Opitz & Zhou, 2012).
FAP / SEPRASE ENSG00000078098	1 (P=0.98) Network member	Seed	Plasma membrane serine protease/gelatinase. FAP has been associated with a mesenchymal phenotype in stromal tumours and depletion of FAP+ cells leads to tumour regression(Kraman et al., 2010). It is expressed on reactive fibroblasts in remodeled tissues and in cultured fibroblasts(Rettig et al., 1993).	
CA12 ENSG00000074410	1 (P=0.98) Network member	Seed	Plasma membrane carbonic anhydrase that regulates local pH via hydration of CO_2 to HCO_3^-.	hyperchlorhidrosis (MIM143860)
**GDF5 / BMP14** ENSG00000125965	1 (P=0.99) Signature member, Network member	Seed	Secreted bone morphogenic protein necessary for skeletal development(Farooq et al., 2013), tendon and ligament development(Dyment et al., 2014), brown fat(Hinoi et al., 2014).	Acromesomelic dysplasia (MIM 201250), Brachydactyly (MIM615072;MIM112600;MIM113100), Chondrodysplasia, (MIM 200700), Du Pan syndrome (MIM22890), Multiple synostoses (MIM 610017), Symphalangism (615298), Osteoarthritis (MIM612400)
**ABI3BP / TARSH** ENSG00000154175	1 (P=1) Signature member, Network member	Seed	Extracellular matrix protein that binds ITGB1. In mouse it regulates the balance between stem cell proliferation/differentiation in bone marrow MSC(Hodgkinson et al., 2013a) and cardiac progenitors (in vitro and in vivo)(Hodgkinson et al., 2013b) . It keeps MSC in a quiescent state (G0/G1)(Hodgkinson et al., 2013a),	Osteochondropathy Kashin-Beck disease (KBD) (Zhang et al., 2014)

			whereas expression of ABI3BP in fibroblasts leads to replicative senescence(Uekawa et al., 2005).	
**CEMIP / KIAA1199** ENSG00000103888	1 (P=1) Signature member	Seed	Hyaluronan-binding and depolymerisation(Yoshida et al., 2013). Secreted on N-terminal processing(Yoshida et al., 2014). Binds EPHA2 (Tiwari et al., 2013). Target of, and regulator of CTTNB1/WNT (Birkenkamp-Demtroder et al., 2011; Evensen et al., 2013; Tiwari et al., 2013). Target of BCL3/NFkB and regulates SEMA3A-dependent EGFR phosphorylation(Shostak et al., 2014) Maintains mesenchymal status after EMT (Evensen et al., 2013; Shostak et al., 2014)	Nonsyndromic deafness (Abe, Usami & Nakamura, 2003; Yoshida et al., 2013)
**ITGA11** ENSG00000137809	1 (P=1) Signature member, Network member	Seed	Collagen receptor with restricted expression on ectomesenchymal cells including myofibroblasts. Regulates PDGFRb-dependent cell migration(Popova et al., 2004) and responds to TGFb signaling in wound healing(Lu et al., 2010). Can enrich CFU-forming MSC from human bone marrow(Kaltz et al., 2010). Expressed in human mesenchymal cells during early skeletal development(Tiger et al., 2001). Mouse Itga11 is necessary for the fusion of muscle satellite cells and inhibition blocks in vitro adipogenesis(Grassot et al., 2014).	No human mutations identified.<br><br>Dwarfism in Itga11 KO mouse links Itga11 to circulating Igf1 levels(Blumbach et al., 2012).
**PRRX1** ENSG00000116132	1 (P=1) Signature member, Network member	Seed	Transcription factor required for mesenchymal lineage development. Necessary for formation of multiple lineages during skeletal development (Martin, Bradley & Olson, 1995). Required for development of vascular smooth muscle(Bergwerff et al., 2000; Jones et al., 2001) and formation of neuroendocrine tissue(Susa et al., 2012). Activates TGFB3 to inhibit adipogenesis(Du et al., 2013). EMT transducer(Ocaña et al., 2012) implicated in a wide range of metastatic carcinomas.	Agnathia-otocephaly complex (OMIM 202650).
APP	Network only	PPI	Interaction partner with SPINT2, BGN, PRR16, CRYAB	Alzheimer disease MIM104300; Cerebral amyloid angiopathy MIM605714 (vascular degeneration).
ACVR2A	Network only	PPI	Interaction partner with GDF5, ENG. Activin type IIA receptor that competitively modulates BMP signaling.	Polymorphisms are associated with early onset pre-eclampsia in several replicated GWAS studies. ACVR2A KO mice model Pierre Robin syndrome (MIM261800) with incomplete penetrance.
BMPR2	Network only	PPI	Interaction partner with CRYAB and GDF5	Pulmonary venoocclusive disease (MIM 265450)
ELN	Network only	PPI	Interaction partner with BGN and EFEMP2	Supravalvar aortic stenosis MIM185500; Cutis laxa, AD MIM 123700
ENG	Network only	PPI	Interaction between ACVR2A, TGFBR1, TGFBR2, TUFM and SP1	MIM187300 hereditary hemorrhagic telangiectasia
GLRX3	Network only	PPI	Interaction partner with VCAM1 and EFEMP2	
HSP90AA1	Network only	PPI	Interaction partner with VCAM1, DDR2 and PDGFRB	
HSPB8	Network only	PPI	Interaction partner with CRYAB and HSPB6	Charcot-Marie-Tooth disease MIM608673
ITGB1	Network only	PPI	Interaction partner with ITGB11 and VCAM1	Fibronectin receptor, embryonic lethal, plays a role in normal organogenesis of many tissues including brain, cartilage, mammary glands and skin.

MDH2	Network only	PPI	Interaction partner with CRYAB and VCAM1	
NEDD4	Network only	PPI	Interaction partner with PDGFR and PRR16	Replicated GWAS associating NEDD4 with Keloid formation, which is a skin fibroproliferative disorder(Nakashima et al., 2010)
NEDD4L	Network only	PPI	Interaction partner with PRR16 and EFEMP2	Regulates TGFb3 signalling by limiting the active half-life of active half-life of SMAD2 and SMAD3(Gao et al., 2009) Regulates ion channel function and 2 polymorphisms are associated with retinopathy MIM603933.
NT5E/CD73	Network only	PPI	Interaction partner with EGR1	MIM211800 Calcification of joints and arteries
PDGFRA	Network only	PPI	Interaction partner with PDGFRB, GRB2, PIK3R1, TGFBR2	MIM606764 Gastrointestinal stromal tumour, MIM191339 isolated cleft palate (pending confirmation)
PPP1CA	Network only	PPI	Interaction partner with VCAM1 and PRR16	Overexpression results in mouse model of dilated cardiomyopathy and endstage heart disease.
PXN	Network only	PPI	Interaction partner with VCAM1 and CCDC80	
SH3KBP1	Network only	PPI	Interaction partner with FAP and PDGFRB	
TGFBR1	Network only	PPI	Interaction partner with BGN and ENG	MIM609192 Loeys-Dietz syndrome, type 1: aortic aneurysm including cleft palate
TGFBR2	Network only	PPI	Interaction partner with PDGFRA and ENG	MIM614331 Colorectal cancer hereditary nonpolyposis. MIM133239 Esophageal cancer, MIM610168 Loeys-Dietz syndrome, type 1: aortic aneurysm including cleft palate
TP53	Network only	PPI	Interaction partner with CRYAB and EFEMP2	Adrenal cortical carcinoma MIM202300;<br>Breast cancer MIM114480; Choroid plexus papilloma MIM260500; Colorectal cancer MIM114500; Hepatocellular carcinoma MIM114550; Li-Fraumeni syndrome MIM151623; Nasopharyngeal carcinoma MIM607107 Osteosarcoma MIM259500; Pancreatic cancer MIM260350; Basal cell carcinoma MIM614740; Glioma susceptibility MIM137800.<br><br>Stabilization of TP53 protein in mouse manifests as CHARGE syndrome (MIM214800) involving coloboma, ear, heart and craniofacial malformations(Van Nostrand et al., 2014).

Supplemental Experimental Procedures

Statistical analysis

Notations

We used the following notations, X denotes the gene expression matrix of n=635 samples and p=27,901 genes and H denotes the number of PLS-components. A class vector Y indicating the class of each sample, categorized as 'MSC' for 125 samples or 'non-MSC' for 510 samples.

A sparse multivariate procedure for discriminant analysis and gene selection

The MSC signature was identified using a novel implementation of the sparse variant of Partial Least Square Discriminant Analysis (sPLS-DA) (Barker and Rayens, 2003; Wold, 1966) implemented for multiple microarray studies using the mixOmics package (Lê Cao et al., 2009)(Lê Cao et al., 2011). sPLS-DA enables the selection of a small subset of genes amongst thousands that best classify the samples into their respective groups (MSC *vs.* non-MCS). It is an iterative multivariate analysis method that seeks a linear combination of the most discriminant genes to maximize the covariance between the gene expression matrix X and the sample vector Y, which is coded as a dummy matrix. The sparsity in the linear combination is achieved through a lasso-type penalization (L1, (Tibshirani, 1996)) and aims to shrink the coefficients associated with non-discriminant genes to zero.

The number of genes to select on each component was estimated by 10-fold stratified cross validation, meaning that each fold contained the same proportion of MSCs over non-MSCs. Each PLS-DA component score is a linear combination of the original gene expression values calculated for each sample. Each sample is projected in the small subspace spanned by the H component scores, allowing for data dimension reduction. The number of components H was chosen according to the criteria defined below, (see "*choosing the number of components*"). The PLS-component scores were used to represent the samples in a PLS-DA scatterplot, and to predict the MSC status of new test samples (Figures 1 and 2).

Constrained sPLS-DA

The standard sPLS-DA method only uses the most discriminative genes to define the PLS-components and needed to be further developed for a cross platform integrative analysis seeking for a robust signature agnostic to microarray platforms. Therefore, we developed a constrained extension of PLS-DA, which defines PLS-components based on specified subsets of stable genes using an internal bootstrap method. The code for this modified implementation is available as a function of BootsPLS R-package (CRAN repository).

The signature tool

Random subsampling

Each random subsampling consisted of splitting the training set (125 MSCs and 510 non-MSCs) into an internal learning set (69% of the data) and an internal test set (31% of the data); each was stratified in order to keep the same proportion of MSCs over non-MSCs. This process was repeated 200 times, to assess the contribution of individual datasets to the stability of the chosen sPLS-DA variables (see Figure 2A of the manuscript).

Cross-Validation

The internal training set was further divided for cross-validation. Following 200 random subsamplings, each of the resulting 200 subsets was further divided using 10-fold cross-validation (CV). A sPLS-DA model was trained on each CV subset and tested on the remaining group of samples to determine the optimal number of genes to select on each four components. We used the Balanced Error Rate (BER), defined as the average of the errors on each class to compensate for the low number of MSC in our training dataset.

Since sPLS-DA is an iterative method that defines each component one at a time, the optimal number of genes selected on a given PLS-DA component is highly dependent on the genes selected on the previous components. To address this component dependency issue we performed a sequential tuning: to tune component h, a constrained sPLS-DA model was built with the genes selected on the (h-1) previous components ($h \leq H$).

Choosing the number of sPLS-DA components

We used the internal classification accuracy as a criterion to evaluate the performance of the sPLS-DA model for a sufficiently large number of components ($H = 6$). A naïve criterion would be to choose the number of components that minimizes the overall classification error rate. However, such criterion would not accommodate the high variability in

the classification error rate across the 200 subsamplings. Instead, we proposed a new criterion that evaluates the gain in classification accuracy when an extra PLS-DA component is added to the sPLS-DA model.

We developed a multiple testing procedure using one-sided t-tests with a null hypothesis H_0^N: '*N* PLS-DA components give better classification accuracy than *N+1* PLS-DA components' ($N \geq 1$) against two alternatives:

H_1^A 'the overall classification accuracy is higher for *N+1* PLS-DA components than for *N* PLS-DA components' and
H_1^B 'the classification accuracy of non-MSC samples only is higher for *N+1* PLS-DA components than for *N* PLS-DA components'

The null hypothesis was rejected when both alternatives H_1^A and H_1^B were true. If H_0^N was rejected - i.e. *N+1* components were considered in the sPLS-DA model, then the null hypothesis was tested for the next component added in the model. We tested the null hypothesis H_0^N for $1 \leq N \leq 5$ or until H_0^N was not rejected. Using the one-sided t-test and the t.test function in R resulted in two p-values for each tested alternative hypothesis. Both p-values were added and the sum was compared to a significance level $\alpha=0.001$ (similar to a Bonferroni correction for each p-value). The reason for this very conservative test was to ensure that the addition of one PLS-DA component would dramatically improve the classification accuracy of the model.

We tested *N* = 5 components, with the associated p-values 1.282936e-58, 7.188488e-25, 3.394169e-10, 3.935352e-03 and 2.816028e-07 respectively. Our final sPLS-DA model therefore included 4 components (Figure 2 of the manuscript).

Choosing the number of stable genes per component
The combination of the 200 subsamplings from the sPLS-DA models enabled us to assess the frequency of selection of each gene (Figure 2A of the manuscript). For each of the four components, we reported the number of times each gene was selected on a given component across the 200 replications, leading to a frequency of selection for each of the 27,901 genes present in the dataset (Figure 2B of the manuscript). We developed an iterative process to accommodate for the gene selection dependency between components and select the best stable gene subset on each PLS-DA component.

Firstly, the genes were assigned a rank according to their decreasing frequency of selection per component, with the same rank given to ties. The top stable genes are identified as most discriminative to differentiate MSC and non-MSC. For each rank *k* going from 1 to *K*, the prediction accuracy of the genes with a rank lower than *k* was assessed with an extra 200 random subsamplings, fitting a constrained sPLS-DA on the most stable genes on the internal learning set and calculating the classification accuracy on the internal test set. We chose *K* = 40, which is a value large enough for our final gene signature.

Secondly, similarly to the method presented to choose the number of components, we developed a multiple testing procedure using one sided t-tests to test the gain in classification accuracy when adding more stable genes to our subset of top ranked stable genes.

Assuming that `$N<M$`, we tested the null hypothesis $H_0^{N,M}$: '*N* top stable genes give a better classification accuracy than *M* top stable genes' against two alternatives:

> H_1^A 'the overall classification is higher for *M* genes than for *N* genes' and
> H_1^B 'the classification accuracy of non-MSC samples is higher for *M* genes than for *N* genes'.

Note that the *N* top stable genes are all included in the *M* top stable genes when $N<M$. We decided to reject the null hypothesis if both alternatives H_1^A and H_1^B were true.

For a fixed *N*, the procedure consisted in successively testing a collection of null hypotheses $\{H_0^{N,M}, M>N\}$ until rejection. If and when a null hypothesis H_0^{N,M^*} was rejected for some M^*, the procedure started again with the collection of null hypotheses $\{H_0^{M^*,R}, R>M^*\}$, where $H_0^{M^*,R}$: 'M^* top stable genes give better classification accuracy than *R* top stable genes'.

The null hypothesis $H_0^{N,M}$ was tested against each alternative H_1^A and H_1^B with a one-sided t-test, resulting in two p-values. Both p-values were added and the sum was compared to a significance level $\alpha=0.01$ (similar to a Bonferroni correction for each p-value). Note that the alternative H_1^C 'the classification accuracy of MSC samples is higher for *M* genes than for *N* genes' was not considered due to the low number of misclassified MSC.

Figure 2C of the manuscript displays the results assessing the top stable genes with respect to our statistical tests for each of the 4 components. Our statistical model selected 5 genes on component 1, 4 genes on component 2, 4 genes on component 3 and 3 genes on component 4, resulting in our final 16-gene signature sPLS-DA model with 4 components.

Definitive assignation of a test sample to the MSC or non-MSC class
To fit our final statistical model, we applied a constrained sPLS-DA on the 16 signature genes identified in our workflow on the full training set (635 samples). Our model led to a prediction score for each external test sample. We derived two quality measures from the prediction scores, namely an uncertainty area and a confidence interval; we combined both to make a definitive prediction call.

From Figure 2E of the manuscript, we determined a cut-off above which 99% of the MSC samples had a prediction score greater than 0.4337, and 99% of the non-MSC samples had a prediction score lower than 0.5169. We defined an 'unsure' area between these two values.

We further assessed the stability of a prediction score by constructing a 95% Confidence Interval (CI) for each test sample. To that end, we recorded a series of prediction scores for each test sample by fitting a constrained sPLS-DA with the 16 signature genes on 200 subsampling of the training set. The 95% CI was then derived from these 200 scores. In all figures where the classification of individual samples is shown, the CI bounds are represented as error bars around each test sample.

From the two quality measures described above, a rule was set to assign each test sample to a MSC, non-MSC or unsure predicted class. A definitive call was made when the 95% CI of a score was not overlapping with the unsure area. A test sample with a prediction score higher than 0.5169 and for which the lower bound of the 95%CI was also higher than 0.5169 was predicted as an MSC. Similarly, a test sample with a prediction score lower than 0.4337 and for which the upper bound of the 95%CI was also lower than 0.4337 was predicted non-MSC class. Any other test samples not falling into these areas were classified as unsure.

Benchmark of the signature
The MSC signature outperforms common MSC surface markers
We investigated the ability of each of the 32 markers defined in Table S1 taken individually to discriminate MSCs vs. non-MSC samples. A PLS-DA method was fitted with each of these 32 single markers on all 635 samples from the training set. The classification accuracy on these samples was recorded using 200 subsamplings (internal learning/test set). Most of these markers (25 out of 32) were reported with a classification error rate of 100%, showing their complete inability to classify the MSCs using our PLS-DA method, with the exception of seven markers CD73, CD105, VCAM1, PDGFRB, KIT, ITGA5 and ANPEP.

The proportion of MSC classified as non-MSC with individual marker expression was: CD73 (18.4%), CD105 (38.4%), VCAM1 (32%), PDGFRB (27.2%), KIT (66.4%), ITGA5 (73.6%), ANPEP (76.8%), and with any of the remaining 25 markers the misclassification was 100%. The proportion of misclassified non-MSC was CD73 (11.2%), CD105 (6%), VCAM1 (6%), PDGFRB (8.24%), KIT (4.9%), ITGA5 (0%), ANPEP (3.53%) and misclassification of non-MSC with the remaining 25 markers was 0%.

Performance of our multivariate approach on the testing set.

External test set	Predicted MSC	Predicted unsure MSC	Predicted non-MSC	Predicted unsure non-MSC	Total
**Potential MSC**	190	2	9	12	213
**Potential non-MSC**	27	3	454	15	499
**Unsure**	191	15	311	62	579
**Total**	408	20	774	89	1291

Batch effect and platform effect
Our 16-gene signature was not confounded by datasets and/or platforms as the sample plot representation from the constrained sPLS-DA in Figure S2 did not show any clusters related to platforms or studies.

REFERENCES

Abe S., Usami SI., Nakamura Y. 2003. Mutations in the gene encoding KIAA1199 protein, an inner-ear protein expressed in Deiters' cells and the fibrocytes, as the cause of nonsyndromic hearing loss. *Journal of Human Genetics* 48:564–570. DOI: 10.1007/s10038-003-0079-2.

Abramsson A., Kurup S., Busse M., Yamada S., Lindblom P., Schallmeiner E., Stenzel D., Sauvaget D., Ledin J., Ringvall M., Landegren U., Kjellén L., Bondjers G., Li JP., Lindahl U., Spillmann D., Betsholtz C., Gerhardt H. 2007. Defective N-sulfation of heparan sulfate proteoglycans limits PDGF-BB binding and pericyte recruitment in vascular development. *Genes and Development* 21:316–331. DOI: 10.1101/gad.398207.

Aghajanova, L., Horcajadas, J.A., Esteban, F.J., and Giudice, L.C. (2010). The bone marrow-derived human mesenchymal stem cell: potential progenitor of the endometrial stromal fibroblast. Biology of reproduction 82, 1076-1087.

Andrade, L.N., Nathanson, J.L., Yeo, G.W., Menck, C.F., and Muotri, A.R. (2012). Evidence for premature aging due to oxidative stress in iPSCs from Cockayne syndrome. Human molecular genetics 21, 3825-3834.

André, T., Meuleman, N., Stamatopoulos, B., De Bruyn, C., Pieters, K., Bron, D., and Lagneaux, L. (2013). Evidences of early senescence in multiple myeloma bone marrow mesenchymal stromal cells. PloS one 8, e59756.

Armulik A., Genové G., Mäe M., Nisancioglu MH., Wallgard E., Niaudet C., He L., Norlin J., Lindblom P., Strittmatter K., Johansson BR., Betsholtz C. 2010. Pericytes regulate the blood-brain barrier. *Nature* 468:557–61. DOI: 10.1038/nature09522.

Barker, M., and Rayens, W. (2003). Partial least squares for discrimination. Journal of chemometrics 17, 166-173.

Beliakova-Bethell, N., Massanella, M., White, C., Lada, S.M., Du, P., Vaida, F., Blanco, J., Spina, C.A., and Woelk, C.H. (2014). The effect of cell subset isolation method on gene expression in leukocytes. Cytometry Part A : the journal of the International Society for Analytical Cytology 85, 94-104.

Benisch, P., Schilling, T., Klein-Hitpass, L., Frey, S.P., Seefried, L., Raaijmakers, N., Krug, M., Regensburger, M., Zeck, S., Schinke, T., et al. (2012). The transcriptional profile of mesenchymal stem cell populations in primary osteoporosis is distinct and shows overexpression of osteogenic inhibitors. PloS one 7, e45142.

Bergwerff M., Gittenberger-de Groot AC., Wisse LJ., DeRuiter MC., Wessels A., Martin JF., Olson EN., Kern MJ. 2000. Loss of function of the Prx1 and Prx2 homeobox genes alters architecture of the great elastic arteries and ductus arteriosus. Virchows Archiv : an international journal of pathology 436:12–19. DOI: 10.1007/PL00008193.

Birkenkamp-Demtroder K., Maghnouj A., Mansilla F., Thorsen K., Andersen CL., Oster B., Hahn S., Orntoft TF. 2011. Repression of KIAA1199 attenuates Wnt-signalling and

decreases the proliferation of colon cancer cells. *British journal of cancer* 105:552–561. DOI: 10.1038/bjc.2011.268.

Blumbach K., Niehoff A., Belgardt BF., Ehlen HWA., Schmitz M., Hallinger R., Schulz J-N., Brüning JC., Krieg T., Schubert M., Gullberg D., Eckes B. 2012. Dwarfism in mice lacking collagen-binding integrins α2β1 and α11β1 is caused by severely diminished IGF-1 levels. *The Journal of biological chemistry* 287:6431–40. DOI: 10.1074/jbc.M111.283119.

Bock, C., Kiskinis, E., Verstappen, G., Gu, H., Boulting, G., Smith, Z.D., Ziller, M., Croft, G.F., Amoroso, M.W., Oakley, D.H., et al. (2011). Reference Maps of human ES and iPS cell variation enable high-throughput characterization of pluripotent cell lines. Cell 144, 439-452.

Bolstad, B.M., Irizarry, R.A., Åstrand, M., and Speed, T.P. (2003). A comparison of normalization methods for high density oligonucleotide array data based on variance and bias. Bioinformatics 19, 185-193.

Bozyk, P.D., Popova, A.P., Bentley, J.K., Goldsmith, A.M., Linn, M.J., Weiss, D.J., and Hershenson, M.B. (2011). Mesenchymal stromal cells from neonatal tracheal aspirates demonstrate a pattern of lung-specific gene expression. Stem cells and development 20, 1995-2007.

Castiglioni, A., Hettmer, S., Lynes, M.D., Rao, T.N., Tchessalova, D., Sinha, I., Lee, B.T., Tseng, Y.H., and Wagers, A.J. (2014). Isolation of progenitors that exhibit myogenic/osteogenic bipotency in vitro by fluorescence-activated cell sorting from human fetal muscle. Stem cell reports 2, 92-106.

Carvalho, B.S., and Irizarry, R.A. (2010). A framework for oligonucleotide microarray preprocessing. Bioinformatics 26, 2363-2367.

Charbord P., Pouget C., Binder H., Dumont F., Stik G., Levy P., Allain F., Marchal C., Richter J., Uzan B., Pflumio F., Letourneur F., Wirth H., Dzierzak E., Traver D., Jaffredo T., Durand C. 2015. A Systems Biology Approach for Defining the Molecular Framework of the Hematopoietic Stem Cell Niche. Cell Stem Cell 15:376–391. DOI: 10.1016/j.stem.2014.06.005.

Chin, M.H., Mason, M.J., Xie, W., Volinia, S., Singer, M., Peterson, C., Ambartsumyan, G., Aimiuwu, O., Richter, L., Zhang, J., et al. (2009). Induced pluripotent stem cells and embryonic stem cells are distinguished by gene expression signatures. Cell stem cell 5, 111-123.

Chung, H.C., Lin, R.C., Logan, G.J., Alexander, I.E., Sachdev, P.S., and Sidhu, K.S. (2012). Human induced pluripotent stem cells derived under feeder-free conditions display unique cell cycle and DNA replication gene profiles. Stem Cells Dev 21, 206-216.

Corsi A., Xu T., Chen XD., Boyde A., Liang J., Mankani M., Sommer B., Iozzo R V., Eichstetter I., Robey PG., Bianco P., Young MF. 2002. Phenotypic effects of biglycan deficiency are linked to collagen fibril abnormalities, are synergized by decorin deficiency, and mimic Ehlers-Danlos-like changes in bone and other connective tissues. *Journal of bone and mineral research : the official journal of the American Society for Bone and Mineral Research* 17:1180–9. DOI: 10.1359/jbmr.2002.17.7.1180.

Crump SM., Abbott GW. 2014. Arrhythmogenic KCNE gene variants: current knowledge and future challenges. *Frontiers in Genetics* 5:3. DOI: 10.3389/fgene.2014.00003.

Dani, N., Olivero, M., Mareschi, K., van Duist, M.M., Miretti, S., Cuvertino, S., Patane, S., Calogero, R., Ferracini, R., Scotlandi, K., et al. (2012). The MET oncogene transforms human primary bone-derived cells into osteosarcomas by targeting committed osteo-progenitors. Journal of bone and mineral research : the official journal of the American Society for Bone and Mineral Research 27, 1322-1334.

de Sousa Andrade, L.N., Nathanson, J.L., Yeo, G.W., Menck, C.F.M., and Muotri, A.R. (2012). Evidence for premature aging due to oxidative stress in iPSCs from Cockayne syndrome. Human molecular genetics, dds211.

Delorme, B., Nivet, E., Gaillard, J., Haupl, T., Ringe, J., Deveze, A., Magnan, J., Sohier, J., Khrestchatisky, M., Roman, F.S., et al. (2010). The human nose harbors a niche of olfactory ectomesenchymal stem cells displaying neurogenic and osteogenic properties. Stem cells and development 19, 853-866.

Doulatov, S., Vo, L.T., Chou, S.S., Kim, P.G., Arora, N., Li, H., Hadland, B.K., Bernstein, I.D., Collins, J.J., Zon, L.I., et al. (2013). Induction of multipotential hematopoietic progenitors from human pluripotent stem cells via respecification of lineage-restricted precursors. Cell Stem Cell 13, 459-470.

Dreiza CM., Komalavilas P., Furnish EJ., Flynn CR., Sheller MR., Smoke CC., Lopes LB., Brophy CM. 2010. The small heat shock protein, HSPB6, in muscle function and disease. *Cell stress & chaperones* 15:1–11. DOI: 10.1007/s12192-009-0127-8.

Du B., Cawthorn WP., Su A., Doucette CR., Yao Y., Hemati N., Kampert S., McCoin C., Broome DT., Rosen CJ., Yang G., Macdougald OA. 2013. The transcription factor paired-related homeobox 1 (Prrx1) inhibits adipogenesis by activating transforming growth factor-β (TGFβ) signaling. *Journal of Biological Chemistry* 288:3036–3047. DOI: 10.1074/jbc.M112.440370.

Du, P., Kibbe, W.A., and Lin, S.M. (2008). lumi: a pipeline for processing Illumina microarray. Bioinformatics 24, 1547-1548.

Dyment NA., Hagiwara Y., Matthews BG., Li Y., Kalajzic I., Rowe DW. 2014. Lineage tracing of resident tendon progenitor cells during growth and natural healing. *PLoS ONE* 9:e96113. DOI: 10.1371/journal.pone.0096113.

Ebert, A.D., Yu, J., Rose, F.F., Mattis, V.B., Lorson, C.L., Thomson, J.A., and Svendsen, C.N. (2009). Induced pluripotent stem cells from a spinal muscular atrophy patient. Nature 457, 277-280.

Espagnolle, N., Guilloton, F., Deschaseaux, F., Gadelorge, M., Sensebe, L., and Bourin, P. (2014). CD146 expression on mesenchymal stem cells is associated with their vascular smooth muscle commitment. Journal of cellular and molecular medicine 18, 104-114.

Erickson LK., Opitz JM., Zhou H. 2012. Lethal Osteogenesis Imperfecta–Like Condition with Cutis Laxa and Arterial Tortuosity in MZ Twins Due to a Homozygous Fibulin-4 Mutation. *Pediatric and Developmental Pathology* 15:137–141. DOI: 10.2350/11-03-1010-CR.1.

Evensen NA., Kuscu C., Nguyen HL., Zarrabi K., Dufour A., Kadam P., Hu YJ., Pulkoski-Gross A., Bahou WF., Zucker S., Cao J. 2013. Unraveling the role of KIAA1199, a novel

endoplasmic reticulum protein, in cancer cell migration. *Journal of the National Cancer Institute* 105:1402–1416. DOI: 10.1093/jnci/djt224.

Farooq M., Nakai H., Fujimoto A., Fujikawa H., Kjaer KW., Baig SM., Shimomura Y. 2013. Characterization of a novel missense mutation in the prodomain of GDF5, which underlies brachydactyly type C and mild Grebe type chondrodysplasia in a large Pakistani family. *Human Genetics* 132:1253–1264. DOI: 10.1007/s00439-013-1330-3.

Frobel, J., Hemeda, H., Lenz, M., Abagnale, G., Joussen, S., Denecke, B., Saric, T., Zenke, M., and Wagner, W. (2014). Epigenetic rejuvenation of mesenchymal stromal cells derived from induced pluripotent stem cells. Stem cell reports *3*, 414-422.

Gagnon-Bartsch, J.A., and Speed, T.P. (2012). Using control genes to correct for unwanted variation in microarray data. Biostatistics 13, 539-552.

Gao S., Alarcón C., Sapkota G., Rahman S., Chen PY., Goerner N., Macias MJ., Erdjument-Bromage H., Tempst P., Massagué J. 2009. Ubiquitin Ligase Nedd4L Targets Activated Smad2/3 to Limit TGF-β Signaling. *Molecular Cell* 36:457–468. DOI: 10.1016/j.molcel.2009.09.043.

Gautier, L., Cope, L., Bolstad, B.M., and Irizarry, R.A. (2004). affy—analysis of Affymetrix GeneChip data at the probe level. Bioinformatics 20, 307-315.

Genbacev, O., Donne, M., Kapidzic, M., Gormley, M., Lamb, J., Gilmore, J., Larocque, N., Goldfien, G., Zdravkovic, T., McMaster, M.T., et al. (2011). Establishment of human trophoblast progenitor cell lines from the chorion. Stem cells 29, 1427-1436.

Gironella M., Malicet C., Cano C., Sandi MJ., Hamidi T., Tauil RMN., Baston M., Valaco P., Moreno S., Lopez F., Neira JL., Dagorn JC., Iovanna JL. 2009. p8/nupr1 regulates DNA-repair activity after double-strand gamma irradiation-induced DNA damage. *Journal of Cellular Physiology* 221:594–602. DOI: 10.1002/jcp.21889.

Gotherstrom, C., Chan, J., O'Donoghue, K., and Fisk, N.M. (2010). Identification of candidate surface antigens for non-invasive prenatal diagnosis by comparative global gene expression on human fetal mesenchymal stem cells. Molecular human reproduction 16, 472-480.

Gottschling, S., Granzow, M., Kuner, R., Jauch, A., Herpel, E., Xu, E.C., Muley, T., Schnabel, P.A., Herth, F.J., and Meister, M. (2013). Mesenchymal stem cells in non-small cell lung cancer--different from others? Insights from comparative molecular and functional analyses. Lung cancer 80, 19-29.

Gottschling, S., Granzow, M., Kuner, R., Jauch, A., Herpel, E., Xu, E.C., Muley, T., Schnabel, P.A., Herth, F.J., and Meister, M. (2013). Mesenchymal stem cells in non-small cell lung cancer—different from others? Insights from comparative molecular and functional analyses. Lung Cancer 80, 19-29.

Granchi, D., Ochoa, G., Leonardi, E., Devescovi, V., Baglìo, S.R., Osaba, L., Baldini, N., and Ciapetti, G. (2009). Gene expression patterns related to osteogenic differentiation of bone marrow–derived mesenchymal stem cells during ex vivo expansion. Tissue Engineering Part C: Methods 16, 511-524.

Grassot V., Da Silva A., Saliba J., Maftah A., Dupuy F., Petit J-M. 2014. Highlights of glycosylation and adhesion related genes involved in myogenesis. *BMC genomics* 15:621. DOI: 10.1186/1471-2164-15-621.

Guenther, M.G., Frampton, G.M., Soldner, F., Hockemeyer, D., Mitalipova, M., Jaenisch, R., and Young, R.A. (2010). Chromatin structure and gene expression programs of human embryonic and induced pluripotent stem cells. Cell Stem Cell 7, 249-257.

Guijarro-Munoz, I., Compte, M., Alvarez-Cienfuegos, A., Alvarez-Vallina, L., and Sanz, L. (2014). Lipopolysaccharide activates Toll-like receptor 4 (TLR4)-mediated NF-kappaB signaling pathway and proinflammatory response in human pericytes. The Journal of biological chemistry 289, 2457-2468.

Hamidouche, Z., Fromigue, O., Ringe, J., Haupl, T., Vaudin, P., Pages, J.C., Srouji, S., Livne, E., and Marie, P.J. (2009). Priming integrin alpha5 promotes human mesenchymal stromal cell osteoblast differentiation and osteogenesis. Proceedings of the National Academy of Sciences of the United States of America 106, 18587-18591.

Hasegawa, R., Tomaru, Y., de Hoon, M., Suzuki, H., Hayashizaki, Y., and Shin, J.W. (2013). Identification of ZNF395 as a novel modulator of adipogenesis. Experimental cell research 319, 68-76.

Hinoi E., Iezaki T., Fujita H., Watanabe T., Odaka Y., Ozaki K., Yoneda Y. 2014. PI3K/Akt is involved in brown adipogenesis mediated by growth differentiation factor-5 in association with activation of the Smad pathway. *Biochemical and biophysical research communications* 450:255–60. DOI: 10.1016/j.bbrc.2014.05.108.

Hirota T., Takahashi A., Kubo M., Tsunoda T., Tomita K., Sakashita M., Yamada T., Fujieda S., Tanaka S., Doi S., Miyatake A., Enomoto T., Nishiyama C., Nakano N., Maeda K., Okumura K., Ogawa H., Ikeda S., Noguchi E., Sakamoto T., Hizawa N., Ebe K., Saeki H., Sasaki T., Ebihara T., Amagai M., Takeuchi S., Furue M., Nakamura Y., Tamari M. 2012. Genome-wide association study identifies eight new susceptibility loci for atopic dermatitis in the Japanese population. *Nature Genetics* 44:1222–1226. DOI: 10.1038/ng.2438.

Hodgkinson CP., Naidoo V., Patti KG., Gomez JA., Schmeckpeper J., Zhang Z., Davis B., Pratt RE., Mirotsou M., Dzau VJ. 2013a. Abi3bp is a multifunctional autocrine/paracrine factor that regulates mesenchymal stem cell biology. *Stem Cells* 31:1669–1682. DOI: 10.1002/stem.1416.

Hodgkinson C., Gomez J., Payne A., Pratt R., Dzau V. 2013b. ABI gene family, member 3 (NESH) binding protein (ABI3BP) regulates cardiac progenitor cell (CPC) differentiation and proliferation. *Circulation* 128.

Hou, R., Yan, H., Niu, X., Chang, W., An, P., Wang, C., Yang, Y., Yan, X., Li, J., and Liu, R. (2014). Gene expression profile of dermal mesenchymal stem cells from patients with psoriasis. Journal of the European Academy of Dermatology and Venereology 28, 1782-1791.

Hu, K., Yu, J., Suknuntha, K., Tian, S., Montgomery, K., Choi, K.D., Stewart, R., Thomson, J.A., and Slukvin, II (2011). Efficient generation of transgene-free induced pluripotent stem cells from normal and neoplastic bone marrow and cord blood mononuclear cells. Blood 117, e109-119.

Jansen, B.J., Gilissen, C., Roelofs, H., Schaap-Oziemlak, A., Veltman, J.A., Raymakers, R.A., Jansen, J.H., Kogler, G., Figdor, C.G., Torensma, R., et al. (2010). Functional differences between mesenchymal stem cell populations are reflected by their transcriptome. Stem cells and development 19, 481-490.

Jarrin M., Pandit T., Gunhaga L. 2012. A balance of FGF and BMP signals regulates cell cycle exit and Equarin expression in lens cells. *Molecular Biology of the Cell* 23:3266–3274. DOI: 10.1091/mbc.E12-01-0075.

Jia, F., Wilson, K.D., Sun, N., Gupta, D.M., Huang, M., Li, Z., Panetta, N.J., Chen, Z.Y., Robbins, R.C., Kay, M.A., et al. (2010). A nonviral minicircle vector for deriving human iPS cells. Nature methods 7, 197-199.

Johnson, W.E., Li, C., and Rabinovic, A. (2007). Adjusting batch effects in microarray expression data using empirical Bayes methods. Biostatistics 8, 118-127.

Jones, M.B., Chu, C.H., Pendleton, J.C., Betenbaugh, M.J., Shiloach, J., Baljinnyam, B., Rubin, J.S., and Shamblott, M.J. (2010). Proliferation and pluripotency of human embryonic stem cells maintained on type I collagen. Stem Cells Dev 19, 1923-1935.

Jones FS., Meech R., Edelman DB., Oakey RJ., Jones PL. 2001. Prx1 controls vascular smooth muscle cell proliferation and tenascin-C expression and is upregulated with Prx2 in pulmonary vascular disease. *Circulation research* 89:131–138. DOI: 10.1161/hh1401.093582.

Kaltz N., Ringe J., Holzwarth C., Charbord P., Niemeyer M., Jacobs VR., Peschel C., Häupl T., Oostendorp RAJ. 2010. Novel markers of mesenchymal stem cells defined by genome-wide gene expression analysis of stromal cells from different sources. *Experimental cell research* 316:2609–17. DOI: 10.1016/j.yexcr.2010.06.002.

Karlsson, K.R., Cowley, S., Martinez, F.O., Shaw, M., Minger, S.L., and James, W. (2008). Homogeneous monocytes and macrophages from human embryonic stem cells following coculture-free differentiation in M-CSF and IL-3. Exp Hematol 36, 1167-1175.

Kawai I., Hisaki T., Sugiura K., Naito K., Kano K. 2012. Discoidin domain receptor 2 (DDR2) regulates proliferation of endochondral cells in mice. *Biochemical and Biophysical Research Communications* 427:611–617. DOI: 10.1016/j.bbrc.2012.09.106.

Kawai I., Matsumura H., Fujii W., Naito K., Kusakabe K., Kiso Y., Kano K. 2014. Discoidin domain receptor 2 (DDR2) regulates body size and fat metabolism in mice. *Transgenic Research* 23:165–175. DOI: 10.1007/s11248-013-9751-2.

Khalid, O., Kim, J.J., Duan, L., Hoang, M., Elashoff, D., and Kim, Y. (2014). Genome-wide transcriptomic alterations induced by ethanol treatment in human dental pulp stem cells (DPSCs). Genomics data 2, 127-131.

Koide Y., Morikawa S., Mabuchi Y., Muguruma Y., Hiratsu E., Hasegawa K., Kobayashi M., Ando K., Kinjo K., Okano H., Matsuzaki Y. 2007. Two distinct stem cell lineages in murine bone marrow. *Stem Cells* 25:1213–1221. DOI: 2006-0325 [pii]\r10.1634/stemcells.2006-0325.

Kokovay E., Wang Y., Kusek G., Wurster R., Lederman P., Lowry N., Shen Q., Temple S. 2012. VCAM1 is essential to maintain the structure of the SVZ niche and acts as an environmental sensor to regulate SVZ lineage progression. *Cell Stem Cell* 11:220–230. DOI: 10.1016/j.stem.2012.06.016.

Konig, J., Huppertz, B., Desoye, G., Parolini, O., Frohlich, J.D., Weiss, G., Dohr, G., Sedlmayr, P., and Lang, I. (2012). Amnion-derived mesenchymal stromal cells show angiogenic properties but resist differentiation into mature endothelial cells. Stem cells and development 21, 1309-1320.

Koyanagi-Aoi, M., Ohnuki, M., Takahashi, K., Okita, K., Noma, H., Sawamura, Y., Teramoto, I., Narita, M., Sato, Y., Ichisaka, T., et al. (2013). Differentiation-defective phenotypes revealed by large-scale analyses of human pluripotent stem cells. Proceedings of the National Academy of Sciences of the United States of America 110, 20569-20574.

Kraman M., Bambrough PJ., Arnold JN., Roberts EW., Magiera L., Jones JO., Gopinathan A., Tuveson DA., Fearon DT. 2010. Suppression of antitumor immunity by stromal cells expressing fibroblast activation protein-alpha. *Science (New York, N.Y.)* 330:827–30. DOI: 10.1126/science.1195300.

Kubo, H., Shimizu, M., Taya, Y., Kawamoto, T., Michida, M., Kaneko, E., Igarashi, A., Nishimura, M., Segoshi, K., Shimazu, Y., et al. (2009). Identification of mesenchymal stem cell (MSC)-transcription factors by microarray and knockdown analyses, and signature molecule-marked MSC in bone marrow by immunohistochemistry. Genes to cells : devoted to molecular & cellular mechanisms 14, 407-424.

Larson, B.L., Ylostalo, J., and Prockop, D.J. (2008). Human multipotent stromal cells undergo sharp transition from division to development in culture. Stem cells 26, 193-201.

Lê Cao, K.-A., Boitard, S., and Besse, P. (2011). Sparse PLS Discriminant Analysis: biologically relevant feature selection and graphical displays for multiclass problems. BMC bioinformatics 12, 253.

Lê Cao, K.-A., González, I., and Déjean, S. (2009). integrOmics: an R package to unravel relationships between two omics datasets. Bioinformatics 25, 2855-2856.

Lê Cao, K.-A., Rohart, F., McHugh, L., Korn, O., and Wells, C.A. (2014). YuGene: A simple approach to scale gene expression data derived from different platforms for integrated analyses. Genomics 103, 239-251.

Lee, E.J., Choi, E.K., Kang, S.K., Kim, G.H., Park, J.Y., Kang, H.J., Lee, S.W., Kim, K.H., Kwon, J.S., Lee, K.H., et al. (2012). N-cadherin determines individual variations in the therapeutic efficacy of human umbilical cord blood-derived mesenchymal stem cells in a rat model of myocardial infarction. Molecular therapy : the journal of the American Society of Gene Therapy 20, 155-167.

Lee, E.S., Chan, J., Shuter, B., Tan, L.G., Chong, M.S., Ramachandra, D.L., Dawe, G.S., Ding, J., Teoh, S.H., Beuf, O., et al. (2009). Microgel iron oxide nanoparticles for tracking human fetal mesenchymal stem cells through magnetic resonance imaging. Stem cells 27, 1921-1931.

Leitinger B. 2014. Discoidin domain receptor functions in physiological and pathological conditions. *International review of cell and molecular biology* 310:39–87. DOI: 10.1016/B978-0-12-800180-6.00002-5.

Leone V., Ferraro A., Schepis F., Federico A., Sepe R., Arra C., Langella C., Palma G., De

Lorenzo C., Troncone G., Masciullo V., Scambia G., Fusco A., Pallante P. 2015. The cl2/dro1/ccdc80 null mice develop thyroid and ovarian neoplasias. *Cancer letters* 357:535–41. DOI: 10.1016/j.canlet.2014.12.010.

Li, R., Ackerman, W.E.t., Summerfield, T.L., Yu, L., Gulati, P., Zhang, J., Huang, K., Romero, R., and Kniss, D.A. (2011). Inflammatory gene regulatory networks in amnion cells following cytokine stimulation: translational systems approach to modeling human parturition. PloS one 6, e20560.

Lim, E., Vaillant, F., Wu, D., Forrest, N.C., Pal, B., Hart, A.H., Asselin-Labat, M.L., Gyorki, D.E., Ward, T., Partanen, A., et al. (2009). Aberrant luminal progenitors as the candidate target population for basal tumor development in BRCA1 mutation carriers. Nat Med 15, 907-913.

Lim, M.N., Hussin, N.H., Othman, A., Umapathy, T., Baharuddin, P., Jamal, R., and Zakaria, Z. (2012). Ex vivo expanded SSEA-4+ human limbal stromal cells are multipotent and do not express other embryonic stem cell markers. Molecular vision 18, 1289.

Lindblom P., Gerhardt H., Liebner S., Abramsson A., Enge M., Hellström M., Bäckström G., Fredriksson S., Landegren U., Nyström HC., Bergström G., Dejana E., Östman A., Lindahl P., Betsholtz C. 2003. Endothelial PDGF-B retention is required for proper investment of pericytes in the microvessel wall. *Genes and Development* 17:1835–1840. DOI: 10.1101/gad.266803.

Loewer, S., Cabili, M.N., Guttman, M., Loh, Y.H., Thomas, K., Park, I.H., Garber, M., Curran, M., Onder, T., Agarwal, S., et al. (2010). Large intergenic non-coding RNA-RoR modulates reprogramming of human induced pluripotent stem cells. Nature genetics 42, 1113-1117.

Loh, Y.H., Hartung, O., Li, H., Guo, C., Sahalie, J.M., Manos, P.D., Urbach, A., Heffner, G.C., Grskovic, M., Vigneault, F., et al. (2010). Reprogramming of T cells from human peripheral blood. Cell Stem Cell 7, 15-19.

Lu N., Carracedo S., Ranta J., Heuchel R., Soininen R., Gullberg D. 2010. The human ??11 integrin promoter drives fibroblast-restricted expression in vivo and is regulated by TGF-??1 in a Smad- and Sp1-dependent manner. *Matrix Biology* 29:166–176. DOI: 10.1016/j.matbio.2009.11.003.

Maherali, N., Ahfeldt, T., Rigamonti, A., Utikal, J., Cowan, C., and Hochedlinger, K. (2008). A high-efficiency system for the generation and study of human induced pluripotent stem cells. Cell Stem Cell 3, 340-345.

Maier, C.L., Shepherd, B.R., Yi, T., and Pober, J.S. (2010). Explant outgrowth, propagation and characterization of human pericytes. Microcirculation 17, 367-380.

Marchetto, M.C., Carromeu, C., Acab, A., Yu, D., Yeo, G.W., Mu, Y., Chen, G., Gage, F.H., and Muotri, A.R. (2010). A model for neural development and treatment of Rett syndrome using human induced pluripotent stem cells. Cell 143, 527-539.

Marchetto, M.C., Yeo, G.W., Kainohana, O., Marsala, M., Gage, F.H., and Muotri, A.R. (2009). Transcriptional signature and memory retention of human-induced pluripotent stem cells. PLoS One 4, e7076.

Markov, V., Kusumi, K., Tadesse, M.G., William, D.A., Hall, D.M., Lounev, V., Carlton, A., Leonard, J., Cohen, R.I., Rappaport, E.F., et al. (2007). Identification of cord blood-derived mesenchymal stem/stromal cell populations with distinct growth kinetics, differentiation potentials, and gene expression profiles. Stem Cells Dev 16, 53-73.

Martin JF., Bradley A., Olson EN. 1995. The paired-like homeo box gene MHox is required for early events of skeletogenesis in multiple lineages. *Genes and Development* 9:1237–1249. DOI: 10.1101/gad.9.10.1237.

Martins, J.P., Santos, J.M., de Almeida, J.M., Filipe, M.A., de Almeida, M.V., Almeida, S.C., Agua-Doce, A., Varela, A., Gilljam, M., Stellan, B., et al. (2014). Towards an advanced therapy medicinal product based on mesenchymal stromal cells isolated from the umbilical cord tissue: quality and safety data. Stem cell research & therapy 5, 9.

Matigian, N., Brooke, G., Zaibak, F., Rossetti, T., Kollar, K., Pelekanos, R., Heazlewood, C., Mackay-Sim, A., Wells, C.A., and Atkinson, K. (2015). Multipotent human stromal cells isolated from cord blood, term placenta and adult bone marrow show distinct differences in gene expression pattern. Genomics Data 3, 70-74.

Menge, T., Zhao, Y., Zhao, J., Wataha, K., Gerber, M., Zhang, J., Letourneau, P., Redell, J., Shen, L., Wang, J., et al. (2012). Mesenchymal stem cells regulate blood-brain barrier integrity through TIMP3 release after traumatic brain injury. Science translational medicine 4, 161ra150.

Morsczeck, C., Schmalz, G., Reichert, T.E., Vollner, F., Saugspier, M., Viale-Bouroncle, S., and Driemel, O. (2009). Gene expression profiles of dental follicle cells before and after osteogenic differentiation in vitro. Clinical oral investigations 13, 383-391.

Mrugala, D., Dossat, N., Ringe, J., Delorme, B., Coffy, A., Bony, C., Charbord, P., Haupl, T., Daures, J.P., Noel, D., et al. (2009). Gene expression profile of multipotent mesenchymal stromal cells: Identification of pathways common to TGFbeta3/BMP2-induced chondrogenesis. Cloning and stem cells 11, 61-76.

Nakashima M., Chung S., Takahashi A., Kamatani N., Kawaguchi T., Tsunoda T., Hosono N., Kubo M., Nakamura Y., Zembutsu H. 2010. A genome-wide association study identifies four susceptibility loci for keloid in the Japanese population. *Nature genetics* 42:768–771. DOI: 10.1038/ng.645.

Nayler, S., Gatei, M., Kozlov, S., Gatti, R., Mar, J.C., Wells, C.A., Lavin, M., and Wolvetang, E. (2012). Induced pluripotent stem cells from ataxia-telangiectasia recapitulate the cellular phenotype. Stem cells translational medicine 1, 523-535.

Novershtern, N., Subramanian, A., Lawton, L.N., Mak, R.H., Haining, W.N., McConkey, M.E., Habib, N., Yosef, N., Chang, C.Y., Shay, T., et al. (2011). Densely interconnected transcriptional circuits control cell states in human hematopoiesis. Cell 144, 296-309.

Van Nostrand JL., Brady C a., Jung H., Fuentes DR., Kozak MM., Johnson TM., Lin C-Y., Lin C-J., Swiderski DL., Vogel H., Bernstein J a., Attié-Bitach T., Chang C-P., Wysocka J., Martin DM., Attardi LD. 2014. Inappropriate p53 activation during development induces features of CHARGE syndrome. *Nature*. DOI: 10.1038/nature13585.

Ocaña OH., Córcoles R., Fabra Á., Moreno-Bueno G., Acloque H., Vega S., Barrallo-Gimeno

A., Cano A., Nieto MA. 2012. Metastatic Colonization Requires the Repression of the Epithelial-Mesenchymal Transition Inducer Prrx1. *Cancer Cell* 22:709–724. DOI: 10.1016/j.ccr.2012.10.012.

Ochi K., Derfoul A., Tuan RS. 2006. A predominantly articular cartilage-associated gene, SCRG1, is induced by glucocorticoid and stimulates chondrogenesis in vitro. *Osteoarthritis and cartilage / OARS, Osteoarthritis Research Society* 14:30–8. DOI: 10.1016/j.joca.2005.07.015.

Ohi, Y., Qin, H., Hong, C., Blouin, L., Polo, J.M., Guo, T., Qi, Z., Downey, S.L., Manos, P.D., Rossi, D.J., et al. (2011). Incomplete DNA methylation underlies a transcriptional memory of somatic cells in human iPS cells. Nat Cell Biol 13, 541-549.

Papadimitropoulos, A., Piccinini, E., Brachat, S., Braccini, A., Wendt, D., Barbero, A., Jacobi, C., and Martin, I. (2014). Expansion of human mesenchymal stromal cells from fresh bone marrow in a 3D scaffold-based system under direct perfusion. PLoS One 9, e102359.

Paquet-Fifield, S., Schluter, H., Li, A., Aitken, T., Gangatirkar, P., Blashki, D., Koelmeyer, R., Pouliot, N., Palatsides, M., Ellis, S., et al. (2009). A role for pericytes as microenvironmental regulators of human skin tissue regeneration. The Journal of clinical investigation 119, 2795-2806.

Patel, J., Seppanen, E., Chong, M.S., Yeo, J.S., Teo, E.Y., Chan, J.K., Fisk, N.M., and Khosrotehrani, K. (2013). Prospective surface marker-based isolation and expansion of fetal endothelial colony-forming cells from human term placenta. Stem cells translational medicine 2, 839-847.

Popova SN., Rodriguez-Sánchez B., Lidén A., Betsholtz C., Van Den Bos T., Gullberg D. 2004. The mesenchymal alpha11beta1 integrin attenuates PDGF-BB-stimulated chemotaxis of embryonic fibroblasts on collagens. *Developmental biology* 270:427–42. DOI: 10.1016/j.ydbio.2004.03.006.

Rajaram, M., Li, J., Egeblad, M., and Powers, R.S. (2013). System-wide analysis reveals a complex network of tumor-fibroblast interactions involved in tumorigenicity. PLoS genetics 9, e1003789.

Rakar, J., Lonnqvist, S., Sommar, P., Junker, J., and Kratz, G. (2012). Interpreted gene expression of human dermal fibroblasts after adipo-, chondro- and osteogenic phenotype shifts. Differentiation; research in biological diversity 84, 305-313.

Rapin, N., Bagger, F.O., Jendholm, J., Mora-Jensen, H., Krogh, A., Kohlmann, A., Thiede, C., Borregaard, N., Bullinger, L., Winther, O., et al. (2014). Comparing cancer vs normal gene expression profiles identifies new disease entities and common transcriptional programs in AML patients. Blood 123, 894-904.

Reinisch, A., Etchart, N., Thomas, D., Hofmann, N., Fruehwirth, M., Sinha, S., Chan, C., Senarath-Yapa, K., Seo, E., and Wearda, T. (2014). Epigenetic and in vivo comparison of diverse MSC sources reveals an endochondral signature for human hematopoietic niche formation. Blood.

Ren G., Zhao X., Zhang L., Zhang J., L'Huillier A., Ling W., Roberts AI., Le AD., Shi S., Shao C., Shi Y. 2010. Inflammatory cytokine-induced intercellular adhesion molecule-1 and vascular cell adhesion molecule-1 in mesenchymal stem cells are critical for immunosuppression. *Journal of Immunology* 184:2321–8. DOI: 10.4049/jimmunol.0902023.

Renard M., Holm T., Veith R., Callewaert BL., Adès LC., Baspinar O., Pickart A., Dasouki M., Hoyer J., Rauch A., Trapane P., Earing MG., Coucke PJ., Sakai LY., Dietz HC., De Paepe AM., Loeys BL. 2010. *Altered TGFbeta signaling and cardiovascular manifestations in patients with autosomal recessive cutis laxa type I caused by fibulin-4 deficiency.* DOI: 10.1038/ejhg.2010.45.

Rettig WJ., Garin-Chesa P., Healey JH., Su SL., Ozer HL., Schwab M., Albino AP., Old LJ. 1993. Regulation and heteromeric structure of the fibroblast activation protein in normal and transformed cells of mesenchymal and neuroectodermal origin. *Cancer research* 53:3327–35.

Royer-Pokora, B., Busch, M., Beier, M., Duhme, C., de Torres, C., Mora, J., Brandt, A., and Royer, H.D. (2010). Wilms tumor cells with WT1 mutations have characteristic features of mesenchymal stem cells and express molecular markers of paraxial mesoderm. Human molecular genetics 19, 1651-1668.

Royer-Zemmour B., Ponsole-Lenfant M., Gara H., Roll P., Lévêque C., Massacrier A., Ferracci G., Cillario J., Robaglia-Schlupp A., Vincentelli R., Cau P., Szepetowski P. 2008. Epileptic and developmental disorders of the speech cortex: Ligand/ receptor interaction of wild-type and mutant SRPX2 with the plasminogen activator receptor uPAR. *Human Molecular Genetics* 17:3617–30. DOI: 10.1093/hmg/ddn256.

Ryan, J.M., Matigian, N., Pelekanos, R.A., Jesuadian, S., Wells, C.A., and Fisk, N.M. (2014). Transcriptional ontogeny of first trimester human fetal and placental mesenchymal stem cells: Gestational age versus niche. Genomics Data 2, 382-385.

Saito, S., Onuma, Y., Ito, Y., Tateno, H., Toyoda, M., Hidenori, A., Nishino, K., Chikazawa, E., Fukawatase, Y., Miyagawa, Y., et al. (2011). Possible linkages between the inner and outer cellular states of human induced pluripotent stem cells. BMC systems biology 5 Suppl 1, S17.

Salomon J., Goulet O., Canioni D., Brousse N., Lemale J., Tounian P., Coulomb A., Marinier E., Hugot J-P., Ruemmele F., Dufier J-L., Roche O., Bodemer C., Colomb V., Talbotec C., Lacaille F., Campeotto F., Cerf-Bensussan N., Janecke AR., Mueller T., Koletzko S., Bonnefont J-P., Lyonnet S., Munnich A., Poirier F., Smahi A. 2014. Genetic characterization of congenital tufting enteropathy: epcam associated phenotype and involvement of SPINT2 in the syndromic form. *Human genetics* 133:299–310. DOI: 10.1007/s00439-013-1380-6.

Sandi MJ., Hamidi T., Malicet C., Cano C., Loncle C., Pierres A., Dagorn JC., Iovanna JL. 2011. p8 Expression controls pancreatic cancer cell migration, invasion, adhesion, and tumorigenesis. *Journal of Cellular Physiology* 226:3442–3451. DOI: 10.1002/jcp.22702.

Schellenberg, A., Joussen, S., Moser, K., Hampe, N., Hersch, N., Hemeda, H., Schnitker, J., Denecke, B., Lin, Q., Pallua, N., et al. (2014). Matrix elasticity, replicative senescence and DNA methylation patterns of mesenchymal stem cells. Biomaterials 35, 6351-6358.

Shao, K., Koch, C., Gupta, M.K., Lin, Q., Lenz, M., Laufs, S., Denecke, B., Schmidt, M., Linke,

M., Hennies, H.C., et al. (2013). Induced pluripotent mesenchymal stromal cell clones retain donor-derived differences in DNA methylation profiles. Molecular therapy : the journal of the American Society of Gene Therapy 21, 240-250.

Shostak K., Zhang X., Hubert P., Göktuna SI., Jiang Z., Klevernic I., Hildebrand J., Roncarati P., Hennuy B., Ladang A., Somja J., Gothot A., Close P., Delvenne P., Chariot A. 2014. NF-κB-induced KIAA1199 promotes survival through EGFR signalling. *Nature communications* 5:5232. DOI: 10.1038/ncomms6232.

Sia GM., Clem RL., Huganir RL. 2013. The human language-associated gene SRPX2 regulates synapse formation and vocalization in mice. *Science (New York, N.Y.)* 342:987–91. DOI: 10.1126/science.1245079.

Si-Tayeb, K., Noto, F.K., Nagaoka, M., Li, J., Battle, M.A., Duris, C., North, P.E., Dalton, S., and Duncan, S.A. (2010). Highly efficient generation of human hepatocyte-like cells from induced pluripotent stem cells. Hepatology 51, 297-305.

Song X., Sato Y., Felemban A., Ito A., Hossain M., Ochiai H., Yamamoto T., Sekiguchi K., Tanaka H., Ohta K. 2012. Equarin is involved as an FGF signaling modulator in chick lens differentiation. *Developmental Biology* 368:109–117. DOI: 10.1016/j.ydbio.2012.05.029.

Song X., Sato Y., Sekiguchi K., Tanaka H., Ohta K. 2013. Equarin is involved in cell adhesion by means of heparan sulfate proteoglycan during lens development. *Developmental Dynamics* 242:23–29. DOI: 10.1002/dvdy.23902.

Spitzer, T.L., Rojas, A., Zelenko, Z., Aghajanova, L., Erikson, D.W., Barragan, F., Meyer, M., Tamaresis, J.S., Hamilton, A.E., Irwin, J.C., et al. (2012). Perivascular human endometrial mesenchymal stem cells express pathways relevant to self-renewal, lineage specification, and functional phenotype. Biology of reproduction 86, 58.

Stunkel, W., Pan, H., Chew, S.B., Tng, E., Tan, J.H., Chen, L., Joseph, R., Cheong, C.Y., Ong, M.L., Lee, Y.S., et al. (2012). Transcriptome changes affecting Hedgehog and cytokine signalling in the umbilical cord: implications for disease risk. PloS one 7, e39744.

Sukarieh, R., Joseph, R., Leow, S.C., Li, Y., Löffler, M., Aris, I.M., Tan, J.H., Teh, A.L., Chen, L., and Holbrook, J.D. (2014). Molecular pathways reflecting poor intrauterine growth are found in Wharton's jelly-derived mesenchymal stem cells. Human Reproduction 29, 2287-2301.

Susa T., Kato T., Yoshida S., Yako H., Higuchi M., Kato Y. 2012. Paired-Related Homeodomain Proteins Prx1 and Prx2 are Expressed in Embryonic Pituitary Stem/Progenitor Cells and May Be Involved in the Early Stage of Pituitary Differentiation. *Journal of Neuroendocrinology* 24:1201–1212. DOI: 10.1111/j.1365-2826.2012.02336.x.

Takahashi, K., Tanabe, K., Ohnuki, M., Narita, M., Sasaki, A., Yamamoto, M., Nakamura, M., Sutou, K., Osafune, K., and Yamanaka, S. (2014). Induction of pluripotency in human somatic cells via a transient state resembling primitive streak-like mesendoderm. Nature communications 5, 3678.

Tanabe, S., Sato, Y., Suzuki, T., Suzuki, K., Nagao, T., and Yamaguchi, T. (2008). Gene expression profiling of human mesenchymal stem cells for identification of novel markers in early- and late-stage cell culture. Journal of biochemistry 144, 399-408.

Tedesco, F.S., Gerli, M.F., Perani, L., Benedetti, S., Ungaro, F., Cassano, M., Antonini, S., Tagliafico, E., Artusi, V., and Longa, E. (2012). Transplantation of genetically corrected human iPSC-derived progenitors in mice with limb-girdle muscular dystrophy. Science translational medicine 4, 140ra189-140ra189.

Teo, A.K., Arnold, S.J., Trotter, M.W., Brown, S., Ang, L.T., Chng, Z., Robertson, E.J., Dunn, N.R., and Vallier, L. (2011). Pluripotency factors regulate definitive endoderm specification through eomesodermin. Genes & development 25, 238-250.

Tibshirani, R. (1996). Regression shrinkage and selection via the lasso. Journal of the Royal Statistical Society Series B (Methodological), 267-288.

Tiger CF., Fougerousse F., Grundström G., Velling T., Gullberg D. 2001. alpha11beta1 integrin is a receptor for interstitial collagens involved in cell migration and collagen reorganization on mesenchymal nonmuscle cells. *Developmental biology* 237:116–129. DOI: 10.1006/dbio.2001.0363.

Tiwari A., Schneider M., Fiorino A., Haider R., Okoniewski MJ., Roschitzki B., Uzozie A., Menigatti M., Jiricny J., Marra G. 2013. Early Insights into the Function of KIAA1199, a Markedly Overexpressed Protein in Human Colorectal Tumors. *PLoS ONE* 8. DOI: 10.1371/journal.pone.0069473.

Tremblay F., Revett T., Huard C., Zhang Y., Tobin JF., Martinez R V., Gimeno RE. 2009. Bidirectional modulation of adipogenesis by the secreted protein Ccdc80/DRO1/URB. *Journal of Biological Chemistry* 284:8136–8147. DOI: 10.1074/jbc.M809535200.

Tremblay F., Huard C., Dow J., Gareski T., Will S., Richard AM., Syed J., Bailey S., Brenneman KA., Martinez R V., Perreault M., Lin Q., Gimeno RE. 2012. Loss of coiled-coil domain containing 80 negatively modulates glucose homeostasis in diet-induced obese mice. *Endocrinology* 153:4290–4303. DOI: 10.1210/en.2012-1242.

Uekawa N., Terauchi K., Nishikimi A., Shimada JI., Maruyama M. 2005. Expression of TARSH gene in MEFs senescence and its potential implication in human lung cancer. *Biochemical and biophysical research communications* 329:1031–1038. DOI: 10.1016/j.bbrc.2005.02.068.

Ullah, M., Sittinger, M., and Ringe, J. (2014). Transdifferentiation of adipogenically differentiated cells into osteogenically or chondrogenically differentiated cells: phenotype switching via dedifferentiation. Int J Biochem Cell Biol 46, 124-137.

Ullah, M., Stich, S., Notter, M., Eucker, J., Sittinger, M., and Ringe, J. (2013). Transdifferentiation of mesenchymal stem cells-derived adipogenic-differentiated cells into osteogenic-or chondrogenic-differentiated cells proceeds via dedifferentiation and have a correlation with cell cycle arresting and driving genes. Differentiation 85, 78-90.

van Gool, S.A., Emons, J.A., Leijten, J.C., Decker, E., Sticht, C., van Houwelingen, J.C., Goeman, J.J., Kleijburg, C., Scherjon, S.A., Gretz, N., et al. (2012). Fetal mesenchymal stromal cells differentiating towards chondrocytes acquire a gene expression profile resembling human growth plate cartilage. PloS one 7, e44561.

Vitale, A.M., Matigian, N., Ravishankar, S., Bellette, B., Wood, S.A., Wolvetang, E., and Mackay-Sim, A. (2012). Variability in the Generation of Induced Pluripotent Stem Cells: Importance for Disease Modeling. Stem Cells Translational Medicine 1, 641-650.

Walenda, G., Abnaof, K., Joussen, S., Meurer, S., Smeets, H., Rath, B., Hoffmann, K., Frohlich, H., Zenke, M., Weiskirchen, R., et al. (2013). TGF-beta1 does not induce senescence of

multipotent mesenchymal stromal cells and has similar effects in early and late passages. PLoS One 8, e77656.

Wold, H. (1966). Multivariate analysis. Academic, New York, 391.

Wu, L., Bluguermann, C., Kyupelyan, L., Latour, B., Gonzalez, S., Shah, S., Galic, Z., Ge, S., Zhu, Y., Petrigliano, F.A., et al. (2013). Human developmental chondrogenesis as a basis for engineering chondrocytes from pluripotent stem cells. Stem cell reports 1, 575-589.

Xu T., Bianco P., Fisher LW., Longenecker G., Smith E., Goldstein S., Bonadio J., Boskey A., Heegaard AM., Sommer B., Satomura K., Dominguez P., Zhao C., Kulkarni AB., Robey PG., Young MF. 1998. Targeted disruption of the biglycan gene leads to an osteoporosis-like phenotype in mice. *Nature genetics* 20:78–82. DOI: 10.1038/1746.

Yamamoto K., Gandin V., Sasaki M., McCracken S., Li W., Silvester JL., Elia AJ., Wang F., Wakutani Y., Alexandrova R., Oo YD., Mullen PJ., Inoue S., Itsumi M., Lapin V., Haight J., Wakeham A., Shahinian A., Ikura M., Topisirovic I., Sonenberg N., Mak TW. 2014. Largen: a molecular regulator of mammalian cell size control. *Molecular cell* 53:904–15. DOI: 10.1016/j.molcel.2014.02.028.

Yoshida H., Nagaoka A., Kusaka-Kikushima A., Tobiishi M., Kawabata K., Sayo T., Sakai S., Sugiyama Y., Enomoto H., Okada Y., Inoue S. 2013. KIAA1199, a deafness gene of unknown function, is a new hyaluronan binding protein involved in hyaluronan depolymerization. *Proceedings of the National Academy of Sciences* 110 :5612–5617. DOI: 10.1073/pnas.1215432110.

Yoshida H., Nagaoka A., Nakamura S., Tobiishi M., Sugiyama Y., Inoue S. 2014. N-Terminal signal sequence is required for cellular trafficking and hyaluronan-depolymerization of KIAA1199. *FEBS letters* 588:111–6. DOI: 10.1016/j.febslet.2013.11.017.

Yu, J., Hu, K., Smuga-Otto, K., Tian, S., Stewart, R., Slukvin, II, and Thomson, J.A. (2009). Human induced pluripotent stem cells free of vector and transgene sequences. Science 324, 797-801.

Zaehres, H., Kogler, G., Arauzo-Bravo, M.J., Bleidissel, M., Santourlidis, S., Weinhold, S., Greber, B., Kim, J.B., Buchheiser, A., Liedtke, S., et al. (2010). Induction of pluripotency in human cord blood unrestricted somatic stem cells. Experimental hematology 38, 809-818, 818 e801-802.

Zhang F., Guo X., Zhang Y., Wen Y., Wang W., Wang S., Yang T., Shen H., Chen X., Tian Q., Tan L., Deng H-W. 2014. Genome-wide copy number variation study and gene expression analysis identify ABI3BP as a susceptibility gene for Kashin–Beck disease. *Human Genetics* 133:793–799. DOI: 10.1007/s00439-014-1418-4.

Zukauskas A., Merley A., Li D., Ang L-H., Sciuto TE., Salman S., Dvorak AM., Dvorak HF., Jaminet S-CS. 2011. TM4SF1: a tetraspanin-like protein necessary for nanopodia formation and endothelial cell migration. *Angiogenesis* 14:345–54. DOI: 10.1007/s10456-011-9218-0.
